# A. Cornelius Celsus

**Published:** 1913-05-24

**Authors:** 


### MEMOS. FROM THE MASTERS.

### A. Cornelius Celsus.

The Rule of Life.

The healthy man, who feeds himself well and has his own free will, needs no binding by rules nor has he any use for physicians or anointers. Let him vary his life, living sometimes in the country, sometimes in the town, and most of all in the open air: let him hunt and sail and take his rest, but most of all exercise, for idleness weakens and labour strengthens the body, the one bringing premature old age, while the other prolongs youthfulness. Also is it well to use a bath, at times of cold water . . . to avoid no common kind of food, to be moderate, but to take food twice a day rather than once, and to take a fair quantity (literally as much as possible) if he can digest it. Such exercise and such diet are proper and therefore athletic exercise is superfluous. To interrupt the order of daily recreation on account of public urgencies is hurtful to the body. He that feeds high grows old soon and is liable to disease. . . . Greater caution is necessary for them that are weak, among such being a great proportion of citizens, and almost all men fond of literature. With these care should rectify that which the nature of the body or excess of study impairs. Of such then he that digests his food well should rise early in the morning, while he that suffers from indigestion should rest longer, or if necessity forces him to get up early he should return to his bed for a longer rest thereafter; he must trust himself to rest entirely, and neither work nor exercise much. He who has crude eructations should drink cold water at intervals; he must live in a well-lighted house, exposed to the breeze in summer and to the sun in winter, avoiding the midday heat, the morning and eventide coolness, and the airs of stagnant pools, nor should he expose himself suddenly to variation of temperature. . . . Nor let him study by candlelight immediately after a meal, but rather wait until part of his food be digested. Let him take suitable, refreshing exercise, leading to perspiration and lassitude, short of actual fatigue, and thereafter rest a while. . . . At his meals let him never overload himself, nor be too abstemious, bearing in mind that intemperance in drink, if intemperance there must be, is safer than intemperance in eating. . . . Both he is in danger who takes food once and he who takes it immoderately. Also is sudden ease after too much labour and sudden labour after too much ease not without danger. When, therefore, any person wishes to alter anything let him do it gradually, accustoming himself to the change. . . . Wherefore in all things ought a man to be moderate and to use discretion in his way of living. (*De Re Medica. Liber I.*)

A Point in the Treatment of Fevers.

The best medicine is food seasonably given. When indeed to give it has been the subject of considerable dispute. Most of the old physicians gave it slowly, on the fifth or sixth day. Asdepides, after harassing his patient for three days with his various measures of treatment allowed food on the fourth day. Themison has of late given food on the third day, withholding it again if the fever returned. . . . There is, however, no general rule. . . . The one thing is that the physician sitting by the bedside should ascertain the strength of the patient. When that is in excess it should be opposed by abstinence. When weakness is to be feared food should be carefully given. For it is the physician's duty neither to weaken the patient by needless abstinence nor load him with superfluous food. (*De Re Medica. Liber III.*)

